# Application of the ADAPT-ITT Model to Develop Trauma-Focused Cognitive Behavioral Therapy for Interpersonal and Racial Trauma and Racial Socialization: Protocol for a Mixed Methods Study

**DOI:** 10.2196/77762

**Published:** 2025-09-18

**Authors:** Isha W Metzger, Sawyer Adams, Karissa Smith, Ashanti Brown, Deron Morrison, Chinelo Aghaji, Liana Giglio, Ashley Ford, Caleb Brown

**Affiliations:** 1 Georgia State University Atlanta United States; 2 University of Georgia Athens, GA United States

**Keywords:** ADAPT-ITT model, Black youth, racial trauma, trauma-focused cognitive behavioral therapy, TF-CBT, racial socialization

## Abstract

**Background:**

Black youth experience higher rates of interpersonal and racial trauma than other youth, yet they are less likely to use and benefit from evidence-based mental health services. These disparities highlight the need for culturally responsive adaptations of existing interventions.

**Objective:**

This study integrated racial socialization (RS)—a protective process for Black youth—into trauma-focused cognitive behavioral therapy (TF-CBT) using the ADAPT-ITT framework (assessment, decision, adaptation, production, topical experts–integration, training, and testing) to improve treatment engagement, acceptability, and outcomes for Black youth and their families.

**Methods:**

Adaptation followed the 8 phases of the ADAPT-ITT model. During *assessment*, a literature review and previous studies identified racial discrimination and socialization as salient risk and protective factors for Black youth, underscoring the need for culturally responsive interventions. In the *decision* phase, TF-CBT was chosen given its evidence base and gaps in addressing racial trauma. The *administration* phase embedded RS messages such as racial pride, barriers, spirituality, and extended family involvement into TF-CBT’s components (psychoeducation, relaxation, affect modulation, cognitive coping, trauma narrative and processing, in vivo mastery, conjoint sessions, and enhancing safety) to enhance psychoeducation, coping, trauma processing, conjoint sessions, and safety planning. In the *production* phase, manuals and fidelity checklists were drafted to preserve TF-CBT’s core elements while embedding RS content. To ensure clinical and cultural relevance, the *topical experts* phase included interviews with 10 Black caregivers and 12 Black youths (aged 12-18 years) who had completed TF-CBT, as well as focus groups with 15 clinicians. Thematic analysis identified adaptations to increase feasibility and acceptability. During *integration*, a racial trauma task force of faculty, graduate, and undergraduate researchers refined the model and produced *The C.A.R.E. Package for Racial Healing*, a culturally informed workbook. In the *training* phase, 28 clinicians from 13 community organizations participated in a Substance Abuse and Mental Health Services Administration–funded learning community, receiving 2 days of training and 12 months of consultation on delivering TF-CBT that integrates RS (TF-CBT-RS). Role-plays, demonstrations, and case discussions supported fidelity and cultural responsiveness. Finally, during *testing*, surveys and feedback indicated that TF-CBT-RS was feasible, acceptable, and associated with improved clinician efficacy, greater treatment engagement, and positive client perceptions of support. Preliminary outcomes suggested reductions in trauma symptoms and improved coping, supporting the need for larger randomized trials.

**Results:**

Integrating RS into TF-CBT enhanced cultural relevance, engagement, and preliminary effectiveness for Black youth and their families.

**Conclusions:**

TF-CBT-RS offers a culturally responsive adaptation of trauma treatment that validates racial stressors, incorporates cultural strengths, and promotes engagement. Broader dissemination of the TF-CBT-RS manual and further testing may reduce disparities in trauma outcomes for Black youth.

**International Registered Report Identifier (IRRID):**

RR1-10.2196/77762

## Introduction

### Black Youth and Trauma

Black youth experience disproportionately high rates of trauma and its negative consequences. While adverse events affect approximately 67% of all youth [1], Black youth are up to 38% more likely than other racial groups to experience interpersonal trauma and polyvictimization**―**defined as exposure to more than one type of traumatic event [2-9]. Such exposure is associated with adverse emotional and behavioral outcomes, including poor mental health [2], lower well-being [10], substance misuse [11,12], and unsafe sexual behaviors [13,14].

In addition to the disparities in trauma exposure, nearly 40% of Black youth face racism and discrimination—directly (eg, being told that their hair is not professional) and indirectly (eg, witnessing police brutality on social media)—which constitute significant sources of stress [15-20]. These experiences may contribute to negative health outcomes similar to those of trauma exposure, such as depression, anxiety, hypertension, heart rate variability, and calcification of arteries [21-28]. Comas-Díaz et al [29] propose that severe and prolonged stress reactions due to racism-related stressors constitute *racial trauma.* Jernigan and Daniel [30] demonstrate that these symptoms can be akin to those of posttraumatic stress disorder (PTSD) as defined by the American Psychiatric Association [31] *Diagnostic and Statistical Manual of Mental Disorders, Fifth Edition* [32,33]. Racial trauma can co-occur with other trauma exposure, compounding the symptoms and their impact [2,11,13,14,30,34,35].

In light of recent and ongoing sociopolitical events such as the COVID-19 pandemic and the heightened visibility of racial injustice, the number of Black youths exposed to interpersonal and racial trauma may be increasing [8,36-42]. This presents a growing health concern for clinicians and caregivers alike, underscoring the need for intervention and treatments that are cognizant of these disparities to address high rates of trauma, both racial and interpersonal, in Black youth.

**Figure 1:**
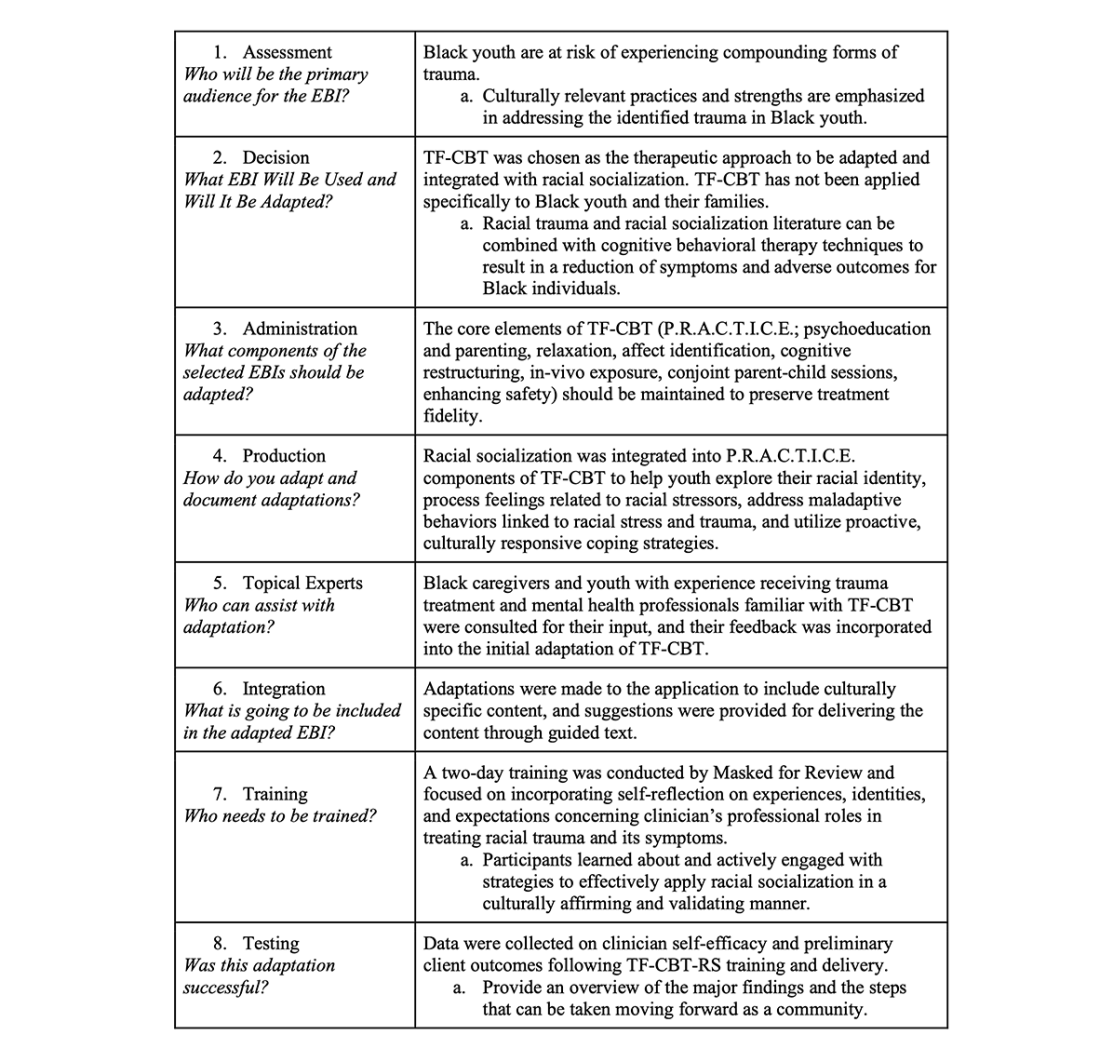
Table 1: ADAPT-ITT Fidelity List.

**Figure 2:**
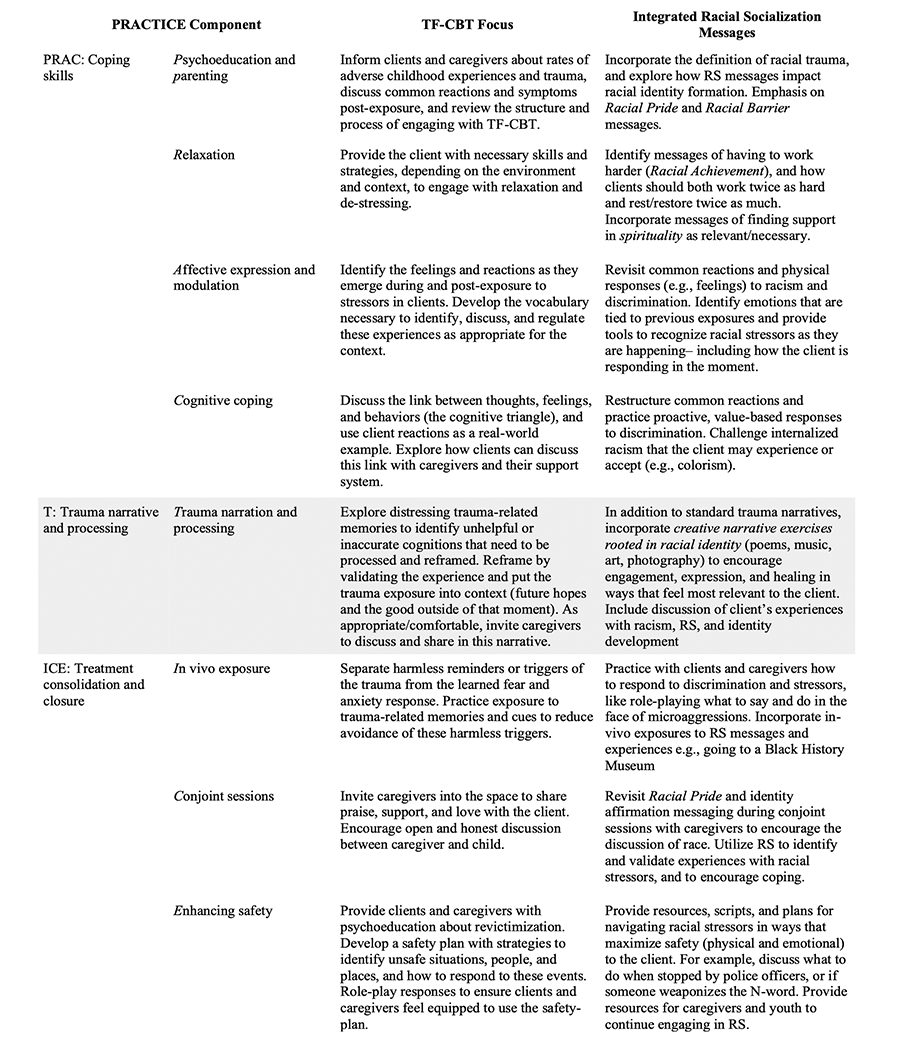
Table 2: TF-CBT with RS Fidelity List.

### Trauma-Focused Cognitive Behavioral Therapy

Evidence-based strategies that address trauma exposure and its consequences for youth do exist, yet Black youth are 3 times less likely to initiate care, be retained in treatment, and remain engaged [19,28,43]. Issues with attrition may be attributed to ongoing systemic issues that deter clients from treatment, such as a lack of diverse practitioners, historical medical racism, and long wait times [44]. Issues with treatment sustainability may also be attributed to the fact that there is often a lack of formal acknowledgment of racial stress and trauma. Without explicit discussion of discrimination (eg, defining microaggressions in an effort to identify them) or culturally relevant coping strategies such as spirituality or creativity, care may feel less relevant or engaging to Black communities [44].

Trauma-focused cognitive behavioral therapy (TF-CBT), for example, is the gold-standard evidence-based trauma treatment for youth aged 3 to 18 years, comprising 8 to 25 sessions [45,46]. This treatment program consists of 8 components: psychoeducation, relaxation, affect modulation, cognitive coping, trauma narrative and processing, in vivo mastery, conjoint sessions, and enhancing safety (PRACTICE). The components are designed to help youth identify stressors and their effects, restructure unsafe or unhelpful responses to stressors, and engage support systems into care to build sustainability. Compared to other clinical treatments for addressing trauma, TF-CBT significantly reduces traumatic stress symptoms and the likelihood of a PTSD diagnosis [45,47,48]. However, despite the treatment’s strengths and efficacy, it does not include culturally specific risk or protective factors for Black youth, such as recognizing racial trauma or discussing common strategies that are used for coping, healing, and navigating racial stressors (eg, preparing for police encounters). This lack of tailored care in TF-CBT—and many other treatment plans—may contribute to the ongoing disparity in the efficacy of treatment and in retaining and engaging Black clients and caregivers in treatment [49-52]. This leaves clinicians to adapt treatment on their own without standardized evidence-based guidance.

Although TF-CBT has been previously adapted for Latinx, Asian American, and American Indian or Alaska Native groups and demonstrated increased therapist self-efficacy and client engagement with the intervention [53-56], none of these adaptations incorporate the experiences of racial stressors and coping specific to Black youth. As such, considering the higher rate of trauma exposure and the compounding nature of interpersonal and racial trauma, it is necessary to develop strength-based treatments that leverage protective factors specific to Black youth [28,53,57].

### Racial Socialization

Racial socialization (RS) is the protective process of transmitting cultural behaviors, attitudes, and values to prepare youth to cope with stressors associated with their ethnic minority status and is associated with positive outcomes, including increased resilience, coping abilities, and decreased problem behaviors and anxiety in Black youth [14,58,59]. There are 6 types of RS messages often represented in the literature, which can be communicated verbally or through modeling relevant behaviors. The most popular socialization messages in the literature are racial barriers, equality, promotion of mistrust, and racial pride [58]. Racial barrier messages prepare youth for instances of racism and discrimination, often by teaching coping mechanisms for dealing with them. Racial equality messages shape children’s attitudes and behaviors toward issues of social justice, discrimination, and inclusivity. Promotion of mistrust aims to caution Black children when interacting with other people from different racial backgrounds. Racial pride messages focus on the positives of one’s racial group, including heritage, history, culture, and the successes of others with a shared identity. These messages can encourage a sense of community and address experiences with racism by helping youth identify the strengths and positives of their racial group. In addition, parents often share messages related to coping through religion or spirituality, finding social support through extended family involvement, and achievement (such as needing to work twice as hard to excel) [1,10,18,60].

Adaptation of treatments to integrate RS as a culturally relevant tool may provide opportunities to increase the efficacy and sustainability of treatment for Black clients. These messages are critical experiences for racially and ethnically marginalized groups who must prepare to face discrimination [61]. Existing interventions that incorporate RS are promising [50,62,63] but have not yet been applied to trauma-focused treatments such as TF-CBT to improve therapists’ self-efficacy, increase client engagement, and directly mitigate the impact of interpersonal and racial trauma for Black youth. There are stand-alone in-person RS interventions [50,64,65]; however, given the benefit of RS in addressing both interpersonal and racial stressors, it is necessary to integrate it into trauma treatments (eg, TF-CBT) for Black youth and do so through evidence-based techniques.

### The ADAPT-ITT Model

The ADAPT-ITT (assessment, decision, adaptation, production, topical experts–integration, training, and testing) model [66] is an evidence-based process for adapting interventions to be more efficacious for marginalized communities. The ADAPT-ITT model is conducted in 8 stages: assessment, decision, administration, production, topical experts, integration, training, and testing. Each phase of the ADAPT-ITT model focuses on maintaining the original evidence-based intervention’s (EBI’s) core elements and theoretical foundations while assessing whether to enhance (eg, provide supplemental materials) or adapt (eg, fundamentally change) aspects of the intervention to better address population-specific needs. The ADAPT-ITT model has been used in many studies, and its repeated success suggests that it is beneficial to use in adapting trauma-focused interventions for racially and ethnically marginalized youth [67-71]. The ADAPT-ITT model provides an optimal framework for clinicians to implement interventions in a culturally sensitive way by calling in population-specific risk and protective factors and ensuring that treatment approaches are most suitable for clients’ cultural backgrounds. Using the ADAPT-ITT model allows for the creation of a formal model for clinicians to consider racism and RS for Black youth in TF-CBT.

### Objective of This Study

TF-CBT and RS have independently been shown to effectively mitigate the negative consequences of trauma, increase youth resilience, and teach healthy coping mechanisms. However, TF-CBT’s lack of systematic consideration and integration of culturally relevant factors (eg, RS techniques) for Black youth and their families may prevent these clients from remaining engaged in and benefiting from treatment. Culturally enhanced treatments may resonate more deeply with Black families and youth by validating cultural identities and normalizing mental health outcomes within these communities. This study acknowledges that Metzger et al [61] were the first to incorporate RS into TF-CBT for Black youth to increase resilience, coping, and communication between youth and their caregivers. Their work has been successfully integrated into a formalized treatment manual [56].

This study builds on the previous work by Metzger et al [56,61] by detailing how the ADAPT-ITT model was used to create a modified application of TF-CBT that integrates RS (TF-CBT-RS) for Black youth and their families and the importance of culturally relevant care for Black clients. The goal and expectation of the adaptation is to boost treatment engagement and use, enhance support for clients experiencing culturally relevant issues (such as racial stress), and reduce associated internalizing and externalizing symptoms. In addition, the adaptation aims to bolster training and support for mental health care providers in culturally competent practice, enabling clinicians to more effectively support Black youth in treatment.

## Methods

### Overview

The ADAPT-ITT model [66] allows for the modification of existing evidence-based treatments by maintaining the integrity of the core elements of a treatment while also addressing and integrating population-specific factors that support the treatment’s relevance, acceptability, and sustainability. This process of adapting existing treatments consists of 8 phases. Table 1 summarizes the 8 steps of the ADAPT-ITT model and how fidelity was maintained within the context of this project.

**Table 1:** ADAPT-ITT (assessment, decision, adaptation, production, topical experts–integration, training, and testing) model steps and significant findings.

Steps	Significant findings
1. Assessment: who will be the primary audience for the EBI^a^?	Black youth are at risk of experiencing compounding forms of trauma. Culturally relevant practices and strengths are emphasized in addressing the identified trauma in Black youth.
2. Decision: what EBI will be used, and will it be adapted?	TF-CBT^b^ was chosen as the therapeutic approach to be adapted and integrated with RS^c^. TF-CBT has not been applied specifically to Black youth and their families. Racial trauma and RS literature can be combined with cognitive behavioral therapy techniques to result in a reduction of symptoms and adverse outcomes for Black individuals.
3. Administration: what components of the selected EBIs should be adapted?	The core elements of TF-CBT (PRACTICE^d^; psychoeducation and parenting, relaxation, affect identification, cognitive restructuring, in-vivo exposure, conjoint parent-child sessions, and enhancing safety) should be maintained to preserve treatment fidelity.
4. Production: how do you adapt and document adaptations?	RS was integrated into PRACTICE components of TF-CBT to help youth explore their racial identity, process feelings related to racial stressors, address maladaptive behaviors linked to racial stress and trauma, and use proactive, culturally responsive coping strategies.
5. Topical experts: who can assist with adaptation?	Black caregivers and youth with experience in receiving trauma treatment and mental health professionals familiar with TF-CBT were consulted for their input, and their feedback was incorporated into the initial adaptation of TF-CBT.
6. Integration: what is going to be included in the adapted EBI?	Adaptations were made to the application to include culturally specific content, and suggestions were provided for delivering the content through guided text.
7. Training: who needs to be trained?	A 2-day training was conducted by masked for review practice and focused on incorporating self-reflection on experiences, identities, and expectations concerning clinicians’ professional roles in treating racial trauma and its symptoms. Participants learned about and actively engaged with strategies to effectively apply RS in a culturally affirming and validating manner.
8. Testing: was this adaptation successful?	Data were collected on clinician self-efficacy and preliminary client outcomes following TF-CBT-RS^e^ training and delivery Provide an overview of the major findings and the steps that can be taken moving forward as a community.

^a^EBI: evidence-based intervention.

^b^TF-CBT: trauma-focused cognitive behavioral therapy.

^c^RS: racial socialization.

^d^PRACTICE: psychoeducation, relaxation, affect modulation, cognitive coping, trauma narrative and processing, in vivo mastery, conjoint sessions, and enhancing safety.

^e^TF-CBT-RS: trauma-focused cognitive behavioral therapy that integrates racial socialization.

### Phase 1 (Assessment): Who Will Be the Primary Audience for the EBI?

Specifically, phase 1 asks researchers to determine the risk and protective processes specific to the target group. The literature identifies Black youth as at risk of experiencing layered and compounding forms of trauma (eg, interpersonal and racial) that need addressing through culturally relevant practices and use of specific cultural strengths (eg, RS). The existing literature shows that Black youth are more likely to be exposed to and experience traumatic events; however, they are 3 times less likely to initiate and complete trauma treatment [19,28,43]. As such, there is a need for new strategies to reach this underserved population. The first author conducted previous studies that explored the role of racial discrimination and socialization as risk and resilience factors in Black youth, demonstrating that there may be a benefit to including RS within clinical treatment to address racial stressors [2,61,72-74]. To address the need for treatment components specific to Black youth, the identified target population was trauma-exposed Black youth. Building on this assessment, the next step involved deciding on the most suitable existing intervention to adapt for this population.

### Phase 2 (Decision): What EBI Will Be Used, and Will It Be Adapted?

As observed in the literature, incorporating culturally specific risk and protective processes within treatment components contributes to improved treatment engagement, sustainability, and efficacy [4,30,37,43,53,64,65,67,68,75]. Due to its recognized effectiveness and recommendation as a treatment for families and children navigating traumatic experiences, TF-CBT was chosen as the therapeutic approach to be adapted and integrated with RS [20,76]. While TF-CBT is an EBI that is effective in addressing externalizing and internalizing trauma-related outcomes and has been previously adapted for specific populations (eg, Latinx and lesbian, gay, bisexual, transgender, and queer populations [53-55]), it has not been adapted and applied in a culturally appropriate manner for Black youth and their families.

As such, it was decided that TF-CBT-RS would be developed to address the needs identified in phase 1. We combined the literature on trauma, racism, and RS with the techniques used in cognitive behavioral therapies. By integrating RS into TF-CBT, Black youth and their families may experience a reduction in the severity and frequency of symptoms and other adverse outcomes associated with experiencing racial trauma. With the intervention identified, the process moved into determining exactly which components of TF-CBT should be adapted to meet the cultural and clinical needs of the target population.

### Phase 3 (Administration): What Components of the Selected EBI Should Be Adapted?

To ensure that the essential mechanisms of change were maintained in the adapted model, researchers ensured fidelity to the original treatment model. Population-specific and necessary culturally specific risk (discrimination) and protective (socialization) mechanisms were applied to each of the PRACTICE components that guide the implementation of TF-CBT. Following the PRACTICE model allows clinicians to support Black clients in gaining the essential psychoeducation and coping skills necessary to later process the discrimination experienced before engaging in important dialogue necessary to restructure their responses to racial stressors to be more value based and proactive [61]. Within the PRACTICE components, RS was operationalized using the 6 most referenced messages in the literature: racial pride, racial barriers, racial equality, achievement, spirituality, and extended family involvement [58]. Table 2 shows how RS was integrated while maintaining fidelity to TF-CBT. These messages were validated through interviews with caregivers and youth that confirmed the relevance of these RS messages to this sample.

RS was integrated into early drafts of the *psychoeducation and parenting* component by providing specific coping strategies and knowledge of discrimination and racial trauma to help youth identify and deal with racial trauma and stressors. Resources were also provided to help caregivers foster a positive sense of racial identity in their Black youth. RS was also integrated to help equip caregivers with the tools to talk about race and racism with the Black youth, educate caregivers on how experiencing racial stressors can impact behavioral outcomes, and identify strategies to help youth build self-esteem. RS was integrated into the *relaxation* component by identifying the cultural messages (eg, “strong Black women don’t ask for help”) and values (the need to work twice as hard as one’s counterparts) that surround the concept of rest and relaxation. For instance, if spirituality was an RS component endorsed by the client, listening to gospel music and prayer were suggested by study participants as possible ways to relax, and *giving thanks* could be used as an exercise in mindfulness. The administration phase allows for suggestions for clients to relax and release their stress in a way that is more accessible and relevant than traditionally taught therapeutic skills.

Within the *affect modulation* component, RS was incorporated to help Black youth identify feelings associated with previous experiences with racial discrimination. For example, youth were guided in scanning their bodies to help them practice attuning themselves to physiological responses to their emotions (described as feelings), such as muscle tension or stomach aches. This provides an opportunity to establish awareness of racial stressors and the psychophysical responses that arise. Integrating RS into the *cognitive coping skills* component helps identify and correct negative, inaccurate, and maladaptive thoughts, emotions, and behaviors following experiences with racial stressors. In this area, youth can be guided to reduce self-blame as a response to racism—such as practicing the thought, “I did not do anything wrong. That person said something racist because that’s their belief, not my *fault*.” Adaptations were also made to provide coping skills toward restructuring clients’ *responses to* racial stressors rather than their *perceptions of* these incidents, as traditional TF-CBT may indicate. This change was because it would be invalidating and inappropriate to imply that a youth’s experience of racism and discrimination is false or not as damaging as it feels to them. Instead, it is more beneficial to ensure that a youth knows that they *did* experience racism and that its effects are real and harmful and encourage control over the situation through proactive and safe responses to the stressor. Specifically, the adaptation to the cognitive coping skills component allows youth to process and role-play proactive (rather than reactive) and value-based techniques in response to hypothetical situations. The *trauma narrative and processing* component integrated RS to support youth in processing their racial trauma–related memories and other symptoms, such as intrusive thoughts, flashbacks, and nightmares. The study team encouraged youth to engage creatively with their experiences of identity and racial stressors. Youth were prompted to express themselves through poetry, music, art, and other outlets to allow for cultural expression and autonomy in creating their own story and understanding of themselves. Moreover, integrating RS into this component provides the opportunity for youth to discuss and understand their feelings regarding their racial identity, cultural traditions, and values and discuss family values and beliefs with regard to responding to racial stressors.

The research team also integrated RS into the *in vivo mastery* component. With the integration of RS, in vivo mastery can help Black youth overcome and address any avoidant behaviors that may have emerged as a response to experiencing racial stressors. In phase 3, developed clinical scripts were designed to use RS by including messages focused on racial pride, spirituality and achievement, traditional forms of social support, and racial barriers and incorporating relevant vocabulary (such as naming racist comments as microaggressions) to help validate and empower youth to approach rather than avoid potentially beneficial situations, experiences, people, and relationships. The *conjoint* *child-parent sessions* component incorporated RS to assist in fostering an environment where youth can openly communicate their racial encounters and experiences of racial stressors with their caregivers. This provides youth and caregivers with a chance to discuss and apply RS messages together and for youth to share successes of RS activities with their caregivers. The final component, *enhancing safety*, used RS to develop culturally responsive safety plans. Families were encouraged to have an open dialogue about potential future racial stressors, for example, how to respond when stopped by police officers, being followed in a store, and being called a racial slur. Incorporating RS into the enhancing safety component may help Black youth and their caregivers identify stressful and potentially harmful situations and prepare them with proactive ways to respond to future racial stressors, such as harassment and microaggressions. After operationalizing RS within TF-CBT’s structure, the next task was to formally document these changes and create a version of the treatment that could be shared, reviewed, and refined.

**Table 2:** Racial socialization (RS) integration in trauma-focused cognitive behavioral therapy (TF-CBT) via psychoeducation, relaxation, affect modulation, cognitive coping, trauma narrative and processing, in vivo mastery, conjoint sessions, and enhancing safety (PRACTICE) stages.

PRACTICE component		TF-CBT focus	Integrated racial socialization messages
**PRAC: coping skills**			
	Psychoeducation and parenting	Inform clients and caregivers about rates of adverse childhood experiences and trauma, discuss common reactions and symptoms after exposure, and review the structure and process of engaging with TF-CBT	Incorporate the definition of racial trauma and explore how RS messages impact racial identity formation; emphasis on *racial pride* and *racial barrier* messages
	Relaxation	Provide the client with necessary skills and strategies, depending on the environment and context, to engage with relaxation and destressing	Identify messages of having to work harder (*racial achievement*) and how clients should both work twice as hard and rest or restore twice as much; incorporate messages of finding support in *spirituality* as relevant or necessary
	Affective expression and modulation	Identify the feelings and reactions as they emerge during and after exposure to stressors in clients; develop the vocabulary necessary to identify, discuss, and regulate these experiences as appropriate for the context	Revisit common reactions and physical responses (eg, feelings) to racism and discrimination; identify emotions that are tied to previous exposures; and provide tools to recognize racial stressors as they are happening, including how the client is responding in the moment
	Cognitive coping	Discuss the link between thoughts, feelings, and behaviors (the cognitive triangle) and use client reactions as a real-world example; explore how clients can discuss this link with caregivers and their support system	Restructure common reactions and practice proactive, value-based responses to discrimination; challenge internalized racism that the client may experience or accept (eg, colorism)
**T: trauma narrative and processing**			
	Trauma narration and processing	Explore distressing trauma-related memories to identify unhelpful or inaccurate cognitions that need to be processed and reframed; reframe by validating the experience and putting the trauma exposure into context (future hopes and the good outside of that moment); invite caregivers to discuss and share in this narrative as appropriate or comfortable	In addition to standard trauma narratives, incorporate creative narrative exercises rooted in racial identity (poems, music, art, and photography) to encourage engagement, expression, and healing in ways that feel most relevant to the client; include discussion of clients’ experiences with racism, RS, and identity development
**ICE: treatment consolidation and closure**			
	In vivo exposure	Separate harmless reminders or triggers of the trauma from the learned fear and anxiety response; practice exposure to trauma-related memories and cues to reduce avoidance of these harmless triggers	Practice with clients and caregivers how to respond to discrimination and stressors, such as role-playing what to say and do in the face of microaggressions; incorporate in-vivo exposures to RS messages and experiences, for example, going to a Black history museum
	Conjoint sessions	Invite caregivers into the space to share praise, support, and love with the client; encourage open and honest discussion between caregiver and child	Revisit racial pride and identity affirmation messaging during conjoint sessions with caregivers to encourage the discussion of race; use RS to identify and validate experiences with racial stressors and encourage coping
	Enhancing safety	Provide clients and caregivers with psychoeducation about revictimization; develop a safety plan with strategies to identify unsafe situations, people, and places, and how to respond to these events; role-play responses to ensure clients and caregivers feel equipped to use the safety plan	Provide resources, scripts, and plans for navigating racial stressors in ways that maximize safety (physical and emotional) to the client, for example, discuss what to do when stopped by police officers or if someone weaponizes the N-word; provide resources for caregivers and youth to continue engaging in RS

### Phase 4 (Production): How Does One Adapt an Intervention and Document Adaptations?

In phase 4, the lead author produced the next version of the existing treatment to maintain fidelity to the TF-CBT model and integrate RS to better address the needs of Black youth. The integration of RS into TF-CBT was documented, and the fidelity checklist outlined by Metzger et al [61] in 2021 was used.

In phases 1 to 4, RS was integrated into TF-CBT by providing a framework to address the unique needs and challenges of Black youth who have been exposed to trauma, particularly racial trauma. TF-CBT-RS can assist clinicians and youth in identifying and modifying maladaptive thoughts, feelings, and behavioral responses associated with the experience of racial trauma. In addition, incorporating RS can enable youth to explore and make sense of their racial identity and address their thoughts regarding their experiences with racial stressors. This was allowed for through the production of the foundational manuscript, “Healing Interpersonal and Racial Trauma” [61]. With a documented adaptation in hand, the project was ready to seek input from experts and those with lived experience to ensure that the intervention was both clinically sound and culturally relevant.

### Ethical Considerations

All study procedures received institutional review board approval (Pro00062769) from the University of Georgia, a Research I institution in the Southeastern United States and were funded by the National Institute of Mental Health from 2016 to 2019. Caregivers and youth were contacted through clinician referrals or flyers, and consent or assent was obtained before participation. Youth, caregivers, and clinicians were compensated for their time, and all identifying information was kept confidential and stored in password-protected files.

## Results

### Phase 5: Topical Experts

To ensure the cultural and clinical relevance of the materials, topical experts were defined as individuals from communities of interest and professionals familiar with the treatment. To aid in the relevance and sustainability of the adapted treatment, we sought out the opinions of individuals who had the lived experience of receiving and delivering trauma treatment (content experts). Specifically, we conducted interviews with 10 Black caregivers (eg, supportive and nonoffending guardians, biological parents, or foster parents of youth) and 12 Black youths (aged 12-18 years) who had previously engaged in TF-CBT due to experiences with interpersonal trauma. We also organized and facilitated focus groups with 15 diverse Master’s- and doctoral-level clinicians familiar with and knowledgeable about the TF-CBT framework and who provided services to Black youth and their caregivers.

Participants were recruited through community-based organizations (ie, children’s advocacy centers) with whom the lead author had ongoing partnerships. Eligibility criteria for caregivers and youth included identifying as Black or African American, having completed or partially completed TF-CBT, and willingness to provide feedback on the treatment adaptation. Caregiver and youth interviews were conducted via Zoom (Zoom Video Communications) and audio recorded. Clinicians were invited to participate in in-person focus groups after indicating interest and eligibility (having been previously trained in TF-CBT and completed at least 3 TF-CBT cases). Recordings were transcribed using Otter.ai (Otter.ai Inc) [25] and uploaded into NVivo (QSR International) [6] for qualitative analysis.

To complete this phase, youth, caregiver, and clinician participants viewed the initial treatment application and gave feedback on how feasible and acceptable implementation might be in a clinical setting. These experts reviewed the TF-CBT-RS manual for content accuracy, developmental appropriateness, and cultural relevance. As context experts, caregivers and youth provided feedback on experiences with TF-CBT and how it could have been improved (eg, adding consideration of racial trauma into progressive muscle relaxation exercises, making images depict Black youth, and restructuring responses to racism rather than reactions to racism). Furthermore, caregivers described their experiences with RS and racial stressors and how they mapped onto each treatment component. Thematic analysis was used to identify patterns in the feedback guided by the 6-phase framework by Braun and Clarke [77]. Two coders independently reviewed the transcripts to develop an initial codebook, and discrepancies were resolved through discussion with a third team member. Emergent themes related to feasibility, cultural relevance, and needed adaptations were synthesized and mapped onto specific TF-CBT components for integration into revisions of the materials [61], and this iterative process enhanced the clinical applicability and cultural sensitivity of the intervention. In addition, the first author worked with content experts in TF-CBT, cognitive behavioral therapy, and RS to incorporate culturally relevant components while maintaining treatment fidelity. These experts reviewed drafts of the initial manuscript and fidelity checklist and contributed to developing the revised intervention. With expert feedback from both lived experience and professional perspectives, the adaptation process then moved into integrating these recommendations into the applied intervention in a systematic and sustainable way.

### Phase 6 (Integration): What Is Going to Be Included in the Applied EBI?

To complete this phase, the first author created a racial trauma task force at a Research I university in the Southeastern United States. This task force consisted of the lead author, 1 doctoral-level licensed clinical faculty member, 3 laboratory staff, 11 graduate students, and 10 members enrolled in undergraduate social science programs. Over 4 months, the graduate students met weekly to receive formal training in TF-CBT. During these weekly meetings, graduate students and the first author discussed RS and racial trauma. They brainstormed how related skills and resources could be integrated into each component of TF-CBT (eg, psychoeducation, relaxation, and cognitive restructuring) based on the findings of the previously collected data with context experts (youth and caregiver clients) in phase 5 (refer to Table 2 for integration).

On the basis of the data from content experts, the task force and first author applied recommendations into a translational material that guided both clinicians and clients in the adapted treatment. The resource, titled *The C.A.R.E. Package for Racial Healing*, is a psychoeducation and skill-based workbook detailing the RS-adapted psychoeducation, relaxation, affect modulation, and cognitive coping components of TF-CBT, which helped guide the final application of TF-CBT-RS. While a resource primarily for clinicians and their clients, this workbook can also be more broadly disseminated to be used by Black youth and their caregivers outside of treatment to encourage healing. To create this resource, the task force collaborated with the first author to incorporate the culturally specific content suggested by Black youth and their caregivers, which included relevant examples and exercises for clients to use under the direction of clinicians and other trusted adults who can provide emotional and tangible support. In addition to providing suggestions on how to effectively use coping strategies via guided text in the *C.A.R.E. Package* workbook, members of the task force worked with a graphic designer to include culturally relevant images and activities in the workbook. Once integration was complete, the next step was to ensure that the clinicians delivering TF-CBT-RS were properly trained to maintain fidelity while effectively engaging with clients in a culturally responsive way.

### Phase 7 (Training): Who Needs to Be Trained?

In 2022 to 2023, the lead author led a 2-day training followed by 12 sessions of ongoing consultation for a Substance Abuse and Mental Health Services Administration–funded learning community composed of 28 practitioners and supervisors currently delivering child-and-family treatment at 13 community-based organizations nationwide. Clinician training focused on understanding how interpersonal and racial stress and trauma impact the lives of Black youth; examining extensive research on the barriers to and facilitators of service use for Black youth, families, and Black adults; emphasizing the protective role of RS in shaping Black youth’s behavioral and mental health outcomes; and making culturally informed decisions on engagement, assessment, and treatment of Black youth. Clinicians were provided with resources and information on interpersonal stressors, racial trauma, and using RS as a culturally specific coping strategy. During these learning community calls, the first author guided clinical practitioners and clinical supervisors working with Black youth and caregivers on the integration of RS into the PRACTICE components of TF-CBT. Clinicians raised questions about incorporating skill-based activities such as role-playing, demonstrations, examples, and client discussions, allowing them to connect their lived experiences with training and further their ability to integrate RS into their delivery of TF-CBT.

In addition, the 2-day training and subsequent sessions provided guidance and opportunities to practice leveraging one’s identity to establish rapport and trust with Black youth, ultimately empowering clinicians to serve as effective allies within the therapeutic space. The training also focused on preparing mental health care providers with the skills and knowledge to successfully carry out the adapted treatment using role-plays, demonstrations, scripts, and ongoing consultation. The training focused on effective techniques and available resources for implementing RS as a cognitive behavioral intervention in a culturally supportive and affirming way for Black youth, families, and adults healing from interpersonal and racial stress and trauma. A significant advancement to support training more culturally competent clinicians is the integration of RS training on the TF-CBT website, which provides national certification for therapists. The manual, which has been widely used by clinicians since its development, is available for download online and offers strategies for implementing TF-CBT and RS (TF-CBT-RS) [56] for Black youth aged 3 to 18 years and their caregivers who experience stress or trauma related to their race or other significant trauma. Once training was completed and clinicians were equipped with the adapted approach, the logical final phase was to test the intervention’s feasibility, clinician efficacy, and preliminary outcomes in real-world practice.

### Phase 8 (Testing): Was the Application Successful?

The first author conducted a series of internet-based learning community sessions focused on TF-CBT-RS over the course of a year. The first author and collaborators (treatment developers Judith Cohen and Anthony Mannarino) gained feedback from practitioners and clinical supervisors who participated in the learning community by delivering a survey following the completion of the learning community to assess feasibility in delivery, therapist efficacy, and the accessibility of the language of the adapted intervention. During the *testing* phase, data were also collected to assess youth outcomes and determine whether TF-CBT-RS successfully reduced youth PTSD symptoms and other mental health problems. Preliminary evidence revealed promising results, including increased clinician efficacy, treatment engagement and use, and client perceptions of support and reduced internalizing and externalizing symptoms [56]. The next steps of testing include conducting a more robust randomized controlled trial to assess the effectiveness of the adapted model in comparison to TF-CBT as typically delivered.

## Discussion

### Expected Findings

This paper describes the process of using the ADAPT-ITT model to integrate RS into TF-CBT to improve treatment engagement and mental health outcomes for Black youth and their families. Although TF-CBT is an effective, evidence-based treatment for trauma-exposed youth aged 3 to 18 years, it does not adequately address the unique impact of racial stressors on Black youth and their families. This paper outlines how the ADAPT-ITT model was used to create TF-CBT-RS, meeting the need for culturally relevant applications of trauma treatment. Such integration and development has the potential to significantly transform the efficacy and sustainability of healing interventions by increasing relevance to, access for, and engagement and retention of Black clients. By using a culturally adapted framework, we propose that TF-CBT-RS will not only increase access for underserved youth but also provide a more comprehensive focus on addressing stressors associated with the lived experiences of Black youth and their families. This work integrates evidence-based tools for mental health treatment—TF-CBT and RS—using a well-validated approach (the ADAPT-ITT model) to address critical areas of risk specific to Black youth. RS is an established cultural strength and presents the opportunity to enhance the utility and delivery of TF-CBT for Black youth and their families who have experienced racial stress and trauma. To address and identify the ways in which racial stress impacts the trauma development of Black youth, it is imperative to research and incorporate the current barriers to and facilitators of such service use for Black youth and their families [56].

During the final phases of development, the first author provided training to multidisciplinary practitioners within a learning community that addressed how to integrate RS strategies into TF-CBT to improve mental health and wellness outcomes. These clinicians received ongoing consultation on the delivery of clinical cases with Black youth who received the integrated treatment and demonstrated promising results. Preliminary findings support the use of RS in TF-CBT, suggesting that this application may improve clinician efficacy and the competence of culturally relevant tools, bolster client perceptions of support, and increase engagement with treatment [50,62-65]. These outcomes also back the use of the ADAPT-ITT model for the adaptation of evidence-based practices for population-specific needs [66-71].

Nonetheless, there remains a need for additional literature to explore how integrating RS into trauma-focused care can improve clinician efficacy, increase client engagement, and improve outcomes related to chronic exposure to racism and discrimination. Larger randomized controlled trials are needed to rigorously compare TF-CBT-RS with standard TF-CBT, examining not only symptom reduction but also engagement, retention, training and implementation, and satisfaction across diverse clinical and community settings.

### Limitations

Although an evidence-based approach was used in making this treatment adaptation and was shown to be beneficial to the participants in the learning community described herein, there are some limitations to consider pertaining to this application of TF-CBT. Clinicians of different experience levels and training backgrounds, as well as youth of varying ages, geographic locations, and cultural identities and their families, may have different perspectives and experiences from those of the participants included in this study. It is important to consider the complexities of Black Americans’ intersecting identities, cultural context, and sociopolitical positioning when assessing feasibility and sustainability. In addition, Black youth who have experienced racial trauma and other forms of trauma, such as sexual abuse, may also respond differently to the adapted treatment. While RS psychoeducation can be modified with examples more relevant to the client themselves, this variability in practice requires oversight to determine its impact on the sustainability and outcomes of the adapted manual—especially as intersectionality (such as lesbian, gay, bisexual, transgender, or queer identity; religion; or immigration status) may influence experience of discrimination and related symptoms, perceptions of support, and the acceptability of the application.

### Conclusions

This study is the first to apply the ADAPT-ITT model to highlight the additive contribution of RS to manualized trauma treatment for youth through a cultural asset lens. The development of more culturally inclusive research in established trauma treatments, greater accessibility to care and facilitation of racial trauma treatment, and more dialogue on the effects of racism in Black communities is a promising future for evidence-based treatment. Integrating RS into prevention and intervention efforts can equip Black youth with the necessary skills to heal from experiences with pervasive racial stressors, which reinforces the need to include cultural assets such as RS in clinical practice. Continued testing of the TF-CBT-RS model can help refine the quality of research and further the value of this work for underserved, ethnically minoritized populations, particularly Black youth and their families.

## Abbreviations

ADAPT-ITT: assessment, decision, adaptation, production, topical experts–integration, training, and testing
EBI: evidence-based intervention
PRACTICE: psychoeducation, relaxation, affect modulation, cognitive coping, trauma narrative and processing, in vivo mastery, conjoint sessions, and enhancing safety
PTSD: posttraumatic stress disorder
RS: racial socialization
TF-CBT: trauma-focused cognitive behavioral therapy
TF-CBT-RS: trauma-focused cognitive behavioral therapy that integrates racial socialization
